# Supplementing Blood Diet With Plant Nectar Enhances Egg Fertility in *Stomoxys calcitrans*

**DOI:** 10.3389/fphys.2021.646367

**Published:** 2021-03-30

**Authors:** Simon K. Tawich, Joel L. Bargul, Daniel Masiga, Merid N. Getahun

**Affiliations:** ^1^International Centre of Insect Physiology and Ecology (icipe), Nairobi, Kenya; ^2^Department of Biochemistry, Jomo Kenyatta University of Agriculture and Technology, Nairobi, Kenya

**Keywords:** *Stomoxys calcitrans*, nectar feeding, insect-plant interaction, fertility, larval emergence

## Abstract

*Stomoxys calcitrans* (stable fly) is a cosmopolitan biting fly of both medical and veterinary importance. Unlike blood-feeding-related behavior of stable fly, its plant feeding, the fitness value, and the *S. calcitrans*–plant interaction are less understood. Here we show based on two chloroplast DNA genes, ribulose bisphosphate carboxylase large chain (*rbcL*) and the intergenic spacer gene *trnH-psbA*, that field-collected male and female stable flies fed on various plant species. We investigated the fitness cost of plant feeding using *Parthenium hysterophorus*, one of the plant species identified to have been fed on by the field-collected flies. Supplementation of blood feeding with a flowering *P. hysterophorus* plant as nectar source enhanced egg hatchability significantly as compared to blood alone, showing the fitness value of nectar supplementation. However, nectar supplementation did not affect the number of eggs laid or longevity of *S. calcitrans* as compared to flies that fed on blood alone. *S. calcitrans* maintained on sugar alone failed to lay eggs. The various plants stable flies fed on demonstrated chemodiversity with their own signature scent. The behavioral response of *S. calcitrans* to these signature compounds varied from strong attraction (γ-terpinene) to neutral (linalool oxide and myrcene) to repellency (butanoic acid). Our study demonstrated that stable flies feed on nectar, and plant nectar supplementation of blood feeding enhanced larval emergence. Thus, our result has implication in stable fly reproduction, survival, disease transmission, boosting laboratory colony, and the possibility of using plant-derived odors for mass trapping of stable fly, for instance, using γ-terpinene.

## Introduction

The stable fly, *Stomoxys calcitrans*, has a global distribution and is one of the most important hematophagous biting flies of livestock (Patra et al., 2018). It is a nuisance fly that inflicts painful bites with serious economic implications due to loss of energy and reduction in yields and spreads various diseases worldwide (Taylor et al., 2012; Getahun et al., 2020b). Insects need a source of macronutrients, carbohydrates, and proteins to support their day-to-day activities, development, and reproduction. However, some hematophagous insects are unique in their dietary requirements and require both sugar and blood for development and reproduction. Unlike mosquitoes, where males exclusively feed on sugar, both male and female stable flies feed on blood from various domestic and wild animals (Mihok and Clausen, 1996; Getahun et al., 2020b). Additionally, field-collected stable flies also consume sugar (Taylor and Berkebile, 2008) and are highly attracted to the fruits and flowers of various plants (Müller et al., 2012) and vinasse, a sugar cane by-product (Jelvez Serra et al., 2017), indicating their plant nectar and sugar feeding behavior. Plant nectar feeding in mosquitoes is well studied and reported to be essential as it increases their lifespan and reproductive capacity and contributes to disease transmission (Foster, 1995; Gary and Foster, 2001; Gu et al., 2011). For example, an increase in pathogen load in mosquitoes has been shown to increase attraction to nectar sources and sugar uptake (Nyasembe et al., 2014) and induce self-medication in honeybees (Simone-Finstrom and Spivak, 2012). Investigating the preferred sugar sources and the role of sugar feeding in stable flies is of great importance to understand their interactions with plants and aid in developing a potential vector control strategy, for instance, through baits. The chemical communication with blood meal sources and/or ovipositional cues using animal-derived odors in stable flies is better studied (Tangtrakulwanich et al., 2015; Baleba et al., 2019; Getahun et al., 2020a). Comparatively, less is known about stable fly–plant interactions, chemodiversity of nectar sources, and fitness cost/benefit of plant nectar feeding. Here, we demonstrate that field-collected stable flies fed on various plants that varied from herbaceous plants to large trees, and plant feeding has fitness benefits. We also show that olfaction plays a key role in the stable flies and plant interaction.

## Materials and Methods

### Study Site

Stable flies were collected from three sampling sites in Kenya: Amboseli – Kajiado County (2.6520° S, 37.2608° E), Mpala Research Centre – Laikipia County (00°23′26.98″N, 036°52′14.98″E), and *icipe* Duduville campus in Nairobi (1°13′12″S, 36°52′48″E). Amboseli and Mpala Research Centre were selected based on the relative abundance of stable flies and availability of domestic and wild animals. These are rural settings inhabited by wildlife and pastoralists who keep large herds of livestock such as sheep, goats, and cattle which are potential bloodmeal hosts of stable flies. These two regions are characterized by semi-arid climatic conditions with diverse and structurally complex vegetation cover, being predominantly shrubs and acacia bushes, while *icipe* Duduville campus is an urban setting. Field trapping of stable flies in these three regions was done in March and April 2019 during short-rain season that resulted in a high abundance of stable flies and plant flowering.

### Collection of Stable Flies and DNA Isolation

Field collection of stable flies was conducted using non-baited Vavoua traps (Mihok et al., 1995). Five Vavoua traps were deployed in each of the three respective study sites to trap stable flies for use in the detection of plant feeding (*n* = 300; 100 flies/site for three sites). The collected flies were immobilized on ice, followed by morphological identification prior to preservation in absolute ethanol in Falcon tubes, and stored at −20°C before DNA extraction was conducted. During the DNA pre-extraction steps, stable flies were dipped in 1% sodium hydroxide for 1 min to remove any exogenous plant material on the insect’s body, and these were rinsed with 1 × phosphate-buffered saline (pH = 7.4) for 1 min. Individual flies were subsequently dissected under a dissecting microscope to isolate the gut contents for genomic DNA extraction.

The DNA extraction process started by placing the dissected midgut contents of each fly in sterilized 1.5-ml Eppendorf tubes, and these were macerated using plastic pestles (Sigma-Aldrich, United States) to lyse the cells. Genomic DNA was extracted from the contents of the midgut using DNeasy blood and tissue kit (Qiagen, Hilden, Germany) following the manufacturer’s protocol. The extracted DNA was eluted in 50 μl of elution buffer, and the concentrations were quantified using a NanoDrop spectrophotometer (Thermo Scientific, Wilmington, DE, United States) by comparing the absorbance at 260 and 280 nm. The isolated DNA samples were stored at −20°C until use.

### PCR Amplification of Chloroplast DNA and Gene Sequencing to Identify the Plant Origin of Nectar Consumed by Flies

A combination of two-gene chloroplast target consisting of coding (rbcL gene) and non-coding gene spacer region (trnH-psbA) was used to identify host plants from which stable flies in the wild acquire nectar from. The two-gene-marker combination provides the necessary universality and species discrimination power (Kress and Erickson, 2007) and therefore recommended as a two-locus global land plant barcode. Plant nectar sources were identified using plant-specific PCR-based approaches targeting the above-mentioned genes followed by DNA sequencing. Gene amplification was conducted using chloroplast universal primers adopted in previous studies (Shaw et al., 2005; Bafeel et al., 2011; Abbasi et al., 2018). The rbcL gene was used as standard barcoding region because of its ease of amplification and universality, and it has been widely adopted in previous studies in plant barcoding as well as phylogenetic studies (Vijayan and Tsou, 2010).

All PCR amplifications were conducted using the Proflex 96-well thermal cycler (Applied Biosystems) in 10-μl reaction volumes consisting of 0.5 μl of each primer (10 μM), 1 μl DNA template, 2 μl of 5 × HOT FIREPol EvaGreen HRM mix (Solis BioDyne, Estonia), and 6 μl nuclease-free water. PCR negative control (nuclease-free water in place of DNA template) and positive (+ve) control (fall army warm larvae fed on maize) were included. The optimized PCR conditions were set as follows: initial denaturation at 95°C (15 min) followed by 35 alternating cycles of 95°C (30 s), primer-specific annealing temperature of 50°C for rbcL2, 53°C for both rbcL-A and trnH-psbA primers, extension at 72°C (1 min), and a final elongation at 72°C (10 min). All reactions ended with a final hold step at 4°C. The primers used are listed in Table 1.

**TABLE 1:** The primers used in gene amplification.

Primer name	Binding site	Sequence (5′–3′)	Target gene	Primer Tm	Primer references
trnH-psbA	Forward Reverse	CGCGCATGGTGGATTCACAATCC GTTATGCATGAACGTAATGCT	Intergenic spacer region	66.4°C 55.5°C	Shaw et al., 2005
rbcL2	Forward Reverse	TATGTAGCTTAYCCMTTAGACCTTTTTGAAGA GCTTCGGCACAAAAKARGAARCGGTCTC	Chloroplast	66.1°C 68.7°C	Abbasi et al., 2018
rbcL-A	Forward Reverse	ATGTCACCACAAACAGAGACTAAAGC GTAAAATCAAGTCCACCRCG	Chloroplast	64.7°C 56.4°C	Bafeel et al., 2011

PCR amplicons were resolved through 1% agarose gel stained with 0.5 μg/ml ethidium bromide. Then, a 100-bp DNA ladder (Solis BioDyne, Estonia) was used to estimate the amplicon sizes. The gel images were visualized under a UV transilluminator with a digital Kodak camera (Gel Logic 200 Imaging System, Kodak, Japan). The DNA bands were excised and cleaned using QIAquick purification kit (Qiagen, Hilden, Germany) following the manufacturer’s protocol. The purified samples were outsourced for Sanger sequencing at Macrogen Inc., Netherlands. The obtained sequences were cleaned, edited, and aligned using Geneious software, with contigs from both the forward and reverse sequences forming a consensus sequence. Host plant identification was done by aligning derived sequences against the GenBank database by the use of NCBI BLAST^1^ search engine.

### Fitness Evaluation of Different Feeding Regimes

#### Stable Flies’ Colony Establishment

After demonstrating the nectar feeding behavior of stable fly, we studied the fitness cost of plant nectar feeding under laboratory conditions. To establish the colony of stable flies, wild *S. calcitrans* were trapped at *icipe* Duduville campus, Nairobi, using unbaited Vavoua traps and morphologically identified using Zumpt taxonomic keys (Zumpt, 1973). The collected stable flies were then transferred into 75 × 60 × 45-cm cages for colony establishment at *icipe* insectary. The flies were maintained at a temperature of 25 ± 1°C and RH 50 ± 5% with a 12:12-h light/dark photoperiod. The flies were fed twice a day (at 0800 and 1,600 h) on warm defibrinated bovine blood obtained from a local slaughterhouse (Choice Meat – Kenya). The blood feeding was provided on a moistened cotton wool on a petri dish. Sheep dung was used as oviposition substrate (Baleba et al., 2019), and pupae that developed were picked using soft forceps and transferred into a separate cage for emergence. The newly emerged flies were provided with blood and new substrate to get more flies to be used in repeated experiments.

#### Plant Nectar Source for Stable Fly Under Laboratory Setup

From the list of plant species identified to be fed on by the stable flies (Table 2), *Parthenium hysterophorus* was used as a source of nectar in the fitness value experiment. The choice of this plant for this experiment was based on its availability and the fact that it can be grown in pots as compared to tall acacia trees. Lastly, it is an invasive plant species with wider adaptability. A confirmatory experiment was set up in the laboratory where 20 newly emerged lab-reared *S. calcitrans* flies were placed in an 18 × 18 × 18-cm cage containing a flowering whole intact *P. hysterophorus* for 24 h, and then total gut DNA was extracted. The confirmation of plant feeding experiment was replicated three times, with each cage having 20 stable flies. The stable flies used for the confirmatory plant feeding experiment as well as survival studies for blood + plant used whole intact *P. hysterophorus*. However, for the oviposition bioassay, the flies were fed on freshly cut *P. hysterophorus* flowers provided daily in the oviposition cups. The freshly cut flowers were used for the oviposition experiment rather than the intact plant because of the small sizes of the oviposition cups. To confirm the plant feeding, PCR amplification was performed on extracted samples, and it confirmed positive for cpDNA. Flowering *P. hysterophorus* was obtained locally at the *icipe* compound, transplanted into portable plastic pots, and placed inside the cage where flies were allowed to feed on it.

**TABLE 2:** List of scientific and common names of plant species that provide sugar source for *Stomoxys calcitrans*.

Plant family	Plant species	Common name
Fabaceae	*Terminalia brownii*	Mururuku
Fabaceae	*Senegalia mellifera*	Blackthorn
Fabaceae	*Vachellia xanthophloea*	Fever tree
Asteraceae	*Parthenium hysterophorus*	Famine-weed, feverweed
Amaryllidaceae	*Allium sativum*	Garlic
Anacardiaceae	*Schinus terebinthifolia*	Brazillian pepper tree
Verbenaceae	*Lantana camara*	Tickberry

#### Oviposition Assay and Larval Development

The oviposition of stable flies was investigated under the following treatments: (Exp. I) blood alone with 28 replicates (Exp. II), blood + 10% glucose with 22 replicates, and (Exp. III) blood + plant, with 27 replicates. In each experiment, the number of eggs was counted daily, and the hatchability rate of eggs was quantified. For experiment III, one gravid female and one mature male from each treatment were isolated and kept in a transparent 200-ml oviposition plastic cup with 50 g of sheep dung and freshly cut *P. hysterophorus*. The cup was covered on top using a white nylon mesh material held with a rubber band to prevent the fly from escaping. Eggs laid per fly were recorded daily throughout the fly’s lifetime per treatment. Stable flies lay eggs in batches, and their eggs are 1 mm in length and 0.2 mm in width, white in color, which provides a good contrast from sheep dung, and having a sausage shape, making them easy to see on sheep dung substrate. The eggs laid were counted manually using a soft forceps with the aid of a magnifying hand lens (10 ×) and placed in a fresh substrate for a maximum of 72 h for the eggs to hatch to larvae or be considered non-viable. However, those flies that fed on *P. hysterophorus* only, distilled water alone, or any of the four glucose concentrations (5, 10, 15, and 20%) died before laying eggs, and thus no oviposition data were recorded.

#### Survival Assay

A total of 20 newly emerged stable flies replicated five times per treatment were kept in a 10 × 10 × 15-cm cage made of 6-mm (thick) perspex clear acrylic plastic sheet (Astariglas^®^, Indonesia). Mortality was recorded daily, and dead flies were removed from the cage. The following nine treatments were tested: 30 ml blood only, blood combined with 10% glucose (ratio 3:1, meaning three parts of blood to one part of 10% glucose), blood and *P. hysterophorus*, *P. hysterophorus* alone, four different glucose concentrations (5, 10, 15, and 20%), and distilled water as a negative control. For the glucose feeding experiment, a dry cotton wool was soaked in 30 ml glucose solution.

### Plant Volatile Collection and Extracts Preparation

After the identification of host plants preferred by the stable flies, we aimed to characterize these plants in their volatilome and study stable fly--plants interaction. Volatile collection was done in the plant’s natural habitats using a sigma-portable asymmetric volatile collection pump^2^. The aerial parts of the intact plant including flowers and leaves were enclosed gently in an air-tight oven bag (Reynolds, Richmond, VA, United States). The super Q adsorbent was attached to a Teflon tube connected to portable field pump and placed inside the oven bag. For head space volatile collection, clean air was pushed at 2.5 L/min, and the pull was set at 2 L/min. The experiment was set to run for 12 h, after which the super Q trapped volatiles were eluted in 300 μl gas chromatography with mass spectrometry (GC–MS)-grade hexane.

We further performed hexane extraction of the identified plant species to obtain plant extracts for use in field experiments. One hundred grams of each plant species consisting of flowers and leaves was weighed and crushed using mortar and pestle. Then, 100 ml of hexane solvent was added as an extractant. The mixture was transferred into a glass beaker, covered using an aluminum foil, and kept in darkness for 12 h, after which the extract was filtered using a filter paper (Whatman, circles, diameter 25 mm) and kept under −80°C until use.

### Chemical Analysis

Volatile analysis was done using HP 6890 GC coupled to 5975 MS (Agilent Technologies, Palo Alto, CA, United States). For the analysis, 1 μl of volatile extract was injected into the GC–MS with the auto sampler (Agilent Technologies). A nonpolar capillary HP column was used to separate the compounds, with helium as the carrier gas at a flow rate of 35 cm per second. The oven temperature was held at 35°C for 5 min and programmed to increase at a rate of 10°C per minute to 280°C; it was held at this maximum temperature for 10 min. The chromatograms acquired were analyzed using Agilent MSD Chemstation, whereby the chemical mass spectral data were compared with the library data (Adams2. L and NIST05a.L). The absolute areas of each constituent chemical compound as calculated by the NIST05a.L software were used to estimate their percentage abundance.

After the correct chemical profile for each plant species was determined and named, the individual plants were assigned to groups corresponding to species and distances between and within groups used to calculate *R* values which range between −1 and 1 for each group comparison. Higher positive *R* value close to 1 represents higher dissimilarity between the groups, whereas lower *R* values imply similarity. One-way ANOSIM was conducted to validate if the visual clustering patterns of similarity in non-metric multidimensional scaling plot (NMDS) was statistically different. NMDS provides unbiased insight into the patterns of chemical associations from the tested plant sources. Plants that clustered together are interpreted to have a similar volatile composition than those that are distant. Clustering together of different plant species shows that the plants have a shared volatile profile. The identity of key chemical volatiles within and between plant species was determined using similarity percentage (SIMPER).

### Behavioral Response of Stable Fly to Plant-Derived Volatile Organic Compounds Under Field Conditions

After identifying the plants on which stable fly fed on, we ask: how do they communicate? Their communication involves a signal (volatile organic compounds) and a behavioral response, a response that has been selected over evolutionary time (Baker, 2014). Thus, we conducted field trapping assays at Mpala Research Centre to test the attractivity of selected plant-derived VOCs for those odors that differentiated the six plants into their distinctive taxonomy and plant predictive odors up to 1% differentiation. As there are many VOCs in the plant, we targeted those that are signature odors that differentiate each plant in the classification for each plant stable fly fed on whenever they are available. Vavoua traps were baited with undiluted synthetic standards of volatile organic compounds or plant extracts from selected plants already identified to be fed on by the stable flies (Table 2). For each trap, 2 ml of treatment was pipetted into the 4 ml vial, and dental roll cotton dispenser (10 × 38 mm; Shanghai Dochem Industries Co., Ltd) was inserted and tied 45 cm above the ground on the metal pole, whereas unbaited Vavoua trap was included as a negative control. All treatments were replicated five times at 100 m apart, and each block was separated by 500 m.

### Statistical Analysis

#### Sample Size Calculation

To understand nectar feeding prevalence under field conditions, we assumed that 3% of stable flies feed on nectar (Jones et al., 1992). The sample size was determined using the formula: $n=\frac{\ln\left(\alpha \right)}{\ln\left(1-p\right)}$. By this assumption at 95% confidence limit, *α* = 0.05 and *p* = 0.03 (probability of nectar fed flies). Thus, *n* = −2.9957 / −0.0305 = 98.35, meaning that a minimum of 98 stable flies need to be randomly sampled to get a better picture of nectar feeding (Cameron and Baldock, 1998).

All statistical analyses were performed using R statistical software, version 3.6.2, and PAST, version 4.02. The number of eggs laid as well as their hatchability followed a normal distribution (Shapiro–Wilk test, *p* > 0.05) and hence subjected to analysis of variance (ANOVA). The means were then separated using Student–Newman–Keuls (SNK) test. NMDS was used to cluster the volatile profile of identified headspace volatiles. The chemical profiles of the identified six plant headspaces were compared using one-way ANOSIM with Bray–Curtis dissimilarity index. The survival probabilities of stable flies under different feeding regimes were analyzed using Kaplan–Meier curve, and the differences in survival between the different groups were compared using the log-rank test. The field behavioral data were analyzed using a generalized linear model with negative binomial error distribution and means separated using the SNK test.

## Results

### Plant Feeding: Field-Collected Stable Flies Fed on Various Plants

**FIGURE 1:**
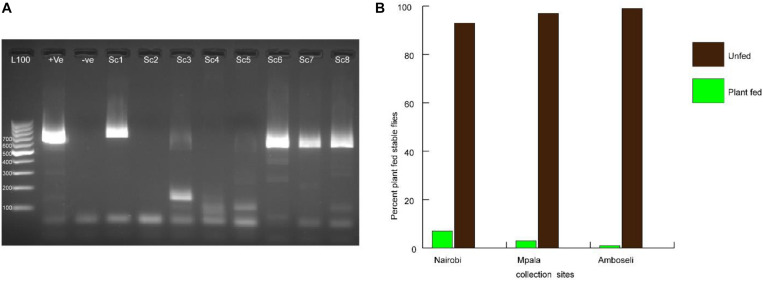
Amplification of target chloroplast marker for identification of plant sources of nectar. **(A)** Electrophoresis of PCR amplicons resolved through 1% agarose gel stained with ethidium bromide for visualization of DNA bands. Positive (+ve) control was fall army warm larvae fed on maize; −ve control was PCR Mastermix with water used as template. Lanes Sc1–Sc8 represent Stomoxys calcitrans DNA samples amplified for chloroplast DNA. **(B)** Graph showing the percent plant-fed stable flies collected from three different sampling sites.

From field-trapped *S. calcitrans*, it was found out that 3.67% of field-collected stable flies had detectable plant material as confirmed by PCR amplification and gene sequencing of plant-specific marker chloroplast regions (Figures 1A,B). From the genomic DNA sequence analysis, the identified plants belonged to the following plant families: *Fabaceae*, *Asteraceae*, *Amaryllidaceae*, *Anacardiaceae*, and *Verbenaceae*. From the five plant families, based on their chloroplast DNA sequence, the following plants were identified as sources of nectar to stable fly from the three sites. In Nairobi, the flies were found positive for *Terminalia brownii* (GenBank accession number MT993360), *Parthenium hysterophorus* (GenBank accession number MT951308), *Lantana camara* (GenBank accession number MT951307), and *Schinus terebinthifolia* (GenBank accession number MT993361). The Mpala collection was positive for *Senegalia mellifera* (GenBank accession number MT993363) and *Vachellia xanthophloea* (GenBank accession number MT993362), whereas the Amboseli catches were only positive for *Allium sativum* (GenBank accession number MT951309) (Table 2).

### Plant Feeding and Biological Cost: Nectar Feeding Supplement Improves Stable Fly Fitness

**FIGURE 2:**
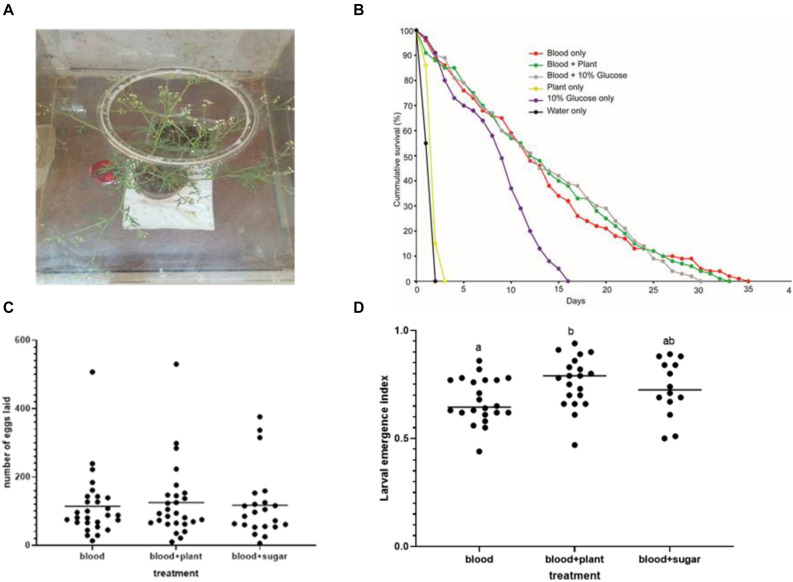
Fitness benefits of sugar feeding in adult stable flies. **(A)** Feeding bioassay, intact flowering Parthenium hysterophorus used as plant nectar source and blood meal in the Petri dish. **(B)** Kaplan–Meier curve showing the survival of adult stable flies when fed under five different feeding regimes including negative (water) and positive control (blood) (log-rank *χ*^2^ test, *n* = 5), *p* < 0.05. **(C)** Number of eggs laid per female stable fly according to the three tested treatments (blood, blood + plant, and blood + sugar). **(D)** Egg hatchability percentage across the three treatments; different letters show significant difference in larval emergence.

The longevity of stable flies did not change when blood feeding was supplemented with *P. hysterophorus* (Figure 2A) nectar feeding as compared to control stable flies fed solely on blood (Figure 2B). Stable flies lived for a maximum of 35 days when maintained on blood alone and 34 days on blood and plant feeding but only survived for 16 days when maintained solely on glucose (Figure 2B). The overall survival analysis using Kaplan–Meier showed that the stable fly’s survival and reproduction were affected differently by the type of food that they feed on, with overall pooled *p*-value = 0.0001. The pairwise comparison using log-rank test using “pairwise_survdiff” function had the following *p*-values: blood alone vs. blood supplemented with glucose (*p* = 0.6), blood vs. blood supplemented with plant nectar (*p* = 0.33), blood + plant vs. blood supplemented with glucose (*p* = 0.24), whereas the difference in survival between the pairs of other tested treatments had *p*-values less than 0.05. Furthermore, even though stable flies lived for 16 days under glucose, they did not lay a single egg. There was no variation in the mean number of eggs laid by the flies under the three different feeding regimes: blood + plant, blood alone, and blood + sugar (ANOVA; df = 2; *F* = 1.082; *p* = 0.346) (Figure 2C). The mean number of eggs laid per female stable fly at 95% confidence interval was 84–167 eggs when fed on blood + plants, 79–149 eggs when solely provided with blood, and 76–160 eggs when supplied with blood + sugar. In some female stable fly, more than 500 eggs were laid per single female (Figure 2C). Even though there was no increase in the mean number of eggs laid, the egg hatchability significantly enhanced when blood feeding was supplemented with plant nectar as compared that with blood alone (ANOVA; df = 2; *F* = 3.82; *p* = 0.0281) (Figure 2D).

### Odor Diversity: Plant Nectar Sources for Stable Flies Exhibited Chemodiversity in Their Semiochemicals

**FIGURE 3:**
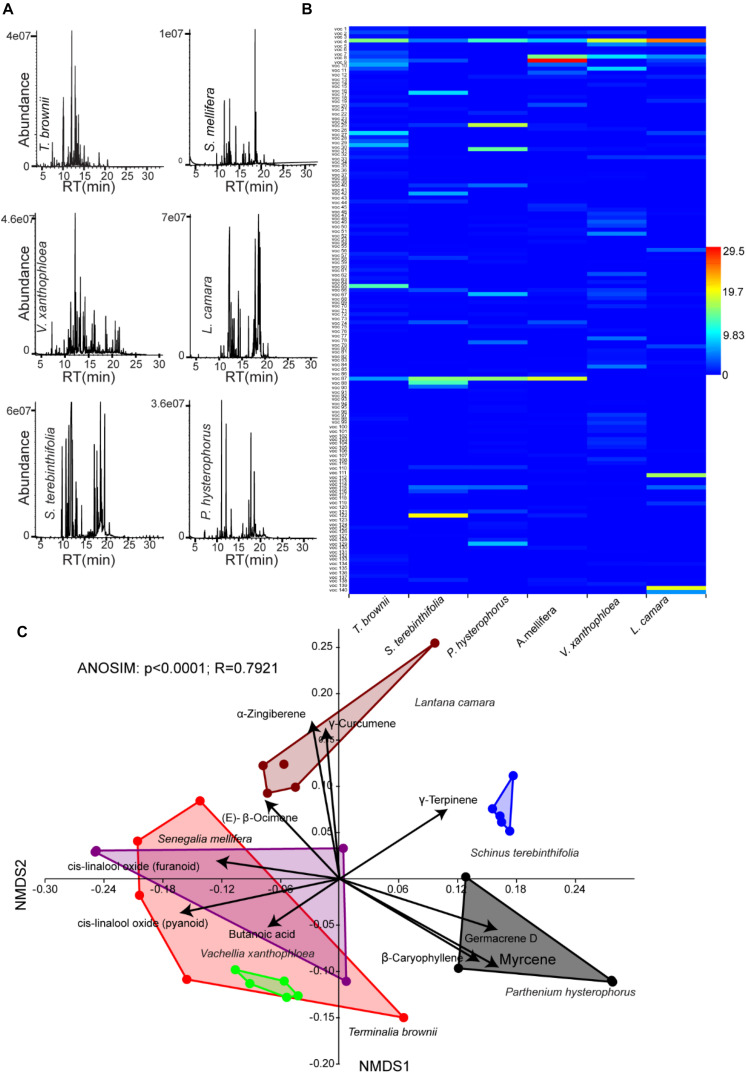
Variations in volatile organic compounds across plant species. **(A)** Representative gas chromatography/mass spectrometry chromatograph of the six plant species including *Terminalia brownii*, *Senegalia mellifera*, *Vachellia xanthophloea*, *Lantana camara*, *Schinus terebinthifolia*, and *Parthenium hysterophorus*. **(B)** Heat map generated from 140 volatile organic compounds (VOCs) (Supplementary Table 1) released from the six plant species which stable flies fed on **(C)**. Multivariate analysis of floral VOCs in six species of plant fed on by stable flies by non-metric multidimensional scaling with a stress value of 0.1448. Black arrows represent overlaid bi-plot analysis of predominant compounds along each axis.

To get insight into the stable flies–plant interaction, we collected head space of volatile organic compounds from the identified six plants that stable fly fed on at the flowering stage (composed of flowers and leaves) and subsequently characterized them using GC–MS (Figure 3A). These plants demonstrated chemodiversity, with about 140 compounds of various chemistry that vary both quantitatively as well as qualitatively between plant species. The scent of the various plants on which stable flies fed on emitted a complex mixture of volatile organic compounds consisting of various terpenes, esters, aldehydes, alcohol, hydrocarbons, and acids (Figure 3B and Supplementary Table 1). The NMDS analysis clustered the six plants into four groups based on their volatile organic compounds (ANOSIM, *p* < 0.0001, with strong correlation, *R* = 0.8, showing that 80% of the separation is explained by the chemodiversity). Interestingly, the three acacia trees (*Senegalia mellifera*, *Vachellia Xanthophloea*, and *T. brownii*) clustered altogether based on their VOCs. However, *L. camara*, *P. hysterophorus*, and *S. terebinthifolia* segregated distinctly between themselves and from the acacia trees. This segregation based on chemistry correlates very well with genomic classification. The three acacia trees with *T. brownii* shared the same signature VOCs, which are (E)-β-ocimene, linalool oxide, and butanoic acid, while the signature compounds of *L. camara* were zingiberene and curcumene and for *P. hysterophorus* were β-caryophyllene, myrcene, and D-germacrene. *S. terebinthifolia* was represented by a single signature compound, γ-terpinene (Figure 3C).

### Behavioral Response of Stable Fly: Stable Fly Is Attracted to Plant-Derived Semiochemicals

**FIGURE 4:**
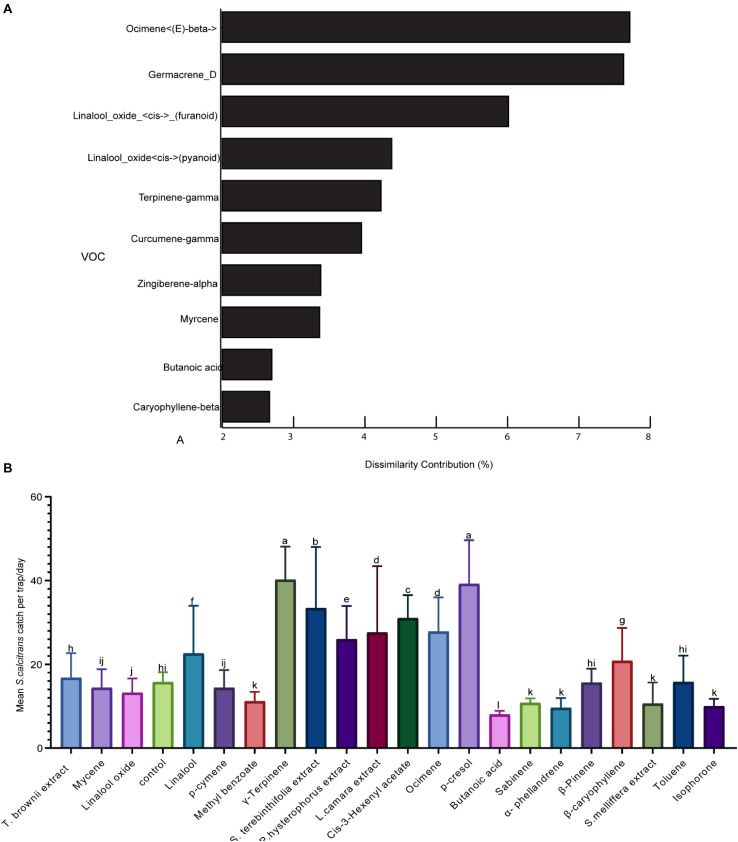
Representation of the 10 most plant-indicative volatile organic compounds (VOCs) across the plant species and field evaluation of their attractivity **(A)**. Similarity percentage analysis and the percent contribution of the predominant compounds for the dissimilarity between the six plant species. **(B)** Graph showing the mean stable flies caught using various VOCs. Bars followed by different letters are statistically different (*p* < 0.05).

To identify the volatile organic compounds that stable flies might use to locate their nectar source plants, we performed behavioral experiments under field conditions. We selected signature VOCs based on SIMPER analysis that determine the relative contribution of different compounds and their availability in the market (Figure 4A). Additional odors that are indicative of plants and contributed up to 1% dissimilarities for their variation between species were also tested (Figures 4A,B). We also added odors such as p-cresol and *cis-*3 hexenyl acetate as positive controls. The predicted signature odor responses varied; some were highly attractive, such as γ-terpinene (the only signature scent of *S. terebinthifolia*) and increased trap catch by × 2.5 as compared to the negative control (Figure 4B). However, most predictive odors were neutral and did not attract more stable flies as compared to the unbaited control. From the plant extracts, *L. camara*, *P. hysterophorus*, and *S. terebinthifolia* attracted significantly more flies as compared to the control (Figure 4B). Butanoic acid, one of the signature scents of the acacias, reduced trap catch by 50% as compared to the negative control. The positive control, p-cresol, and *cis-*3 hexenyl acetate attracted significantly more stable flies as compared to the unbaited control (Figure 4B).

## Discussion

Here, we demonstrate that the stable fly, a known vector of various livestock pathogens, interacts and feeds on various plants. We identified the host plants on which stable fly fed upon in nature from the habitats of this vector using chloroplast DNA sequence accepted marker for plant barcoding. With our life table experiment, we show that plant feeding has fitness benefit for stable flies, as it improves the eggs’ fertility. Stable flies use plant-derived odors to locate their plant nectar source.

A key observation from our work is that supplementing bloodmeal with plant nectar has demonstrated fitness benefit to stable fly by enhancing larval emergence as compared to that of blood alone. This might show that the female stable fly acquired additional resources (i.e., sugars, fats, and amino acids from the plant nectar) that is carried over in stable fly ovaries, which might contribute in the later stages of eggs development and hatching, and such phenomena have been demonstrated in other species as the scarcity of nutrients blocks growth and development (Koenig, 1982; Rivero and Casas, 1999). Furthermore, various plant phytochemicals affect the development of eggs either positively or negatively (Hilker and Meiners, 2011).

Our study demonstrated that supplementing blood diet with *P. hysterophorus* nectar feeding did not change the number of eggs laid or longevity. Similarly, in other insects, plant feeding did not change the fecundity in some but influenced that in others (Kéry et al., 2000; Manda et al., 2007). Feeding on plant nectars and pollen might have diverse advantages for insects such as antifungal, antibacterial, and antiviral, phytochemicals, nutrition as well as energy source (Manson et al., 2010; Koch et al., 2019; Barredo and DeGennaro, 2020) but can also be a source of pathogens, for example, fungal infection affecting the feeding and survival of *Anopheles gambiae* (Manson et al., 2010). Furthermore, nectar constituents such as carbohydrate are known to influence, for instance, mosquito sugar meal choices and longevity both positively and negatively or potentially toxic secondary metabolites such as alkaloids (Ignell et al., 2010; Kessler et al., 2013). Plant sugar feeding also has neutralization benefit of plant defense (Malka et al., 2020). However, we cannot rule out that feeding on plants will not affect either the number of eggs nor survival if we try other plants that *S. calcitrans* has a choice of under natural conditions, as plant species vary in the amount and quality of resources (Roulston et al., 2000; Nyasembe et al., 2020). Most plant nectars found in *A. gambiae* habitats contain sucrose and/or its hexose hydrolysis products, glucose and fructose. The sugar concentration from field plants varies from 8 to 40% (Wykes, 1953). Regarding this, we demonstrated that higher sugar concentration by 10% and above prolonged the longevity of stable flies to 16 days as compared to 5%, which sustained them only for 10 days.

The high mortality of stable flies when fed on *P. hysterophorus* alone shows that this plant might have toxic metabolites that impact the survival and the lack of additional nutrients required for growth and development. Similarly, in other related studies, mosquitoes feeding on some plant species alone resulted in high mortality. Similarly, one night of feeding on the branches of *R. communis*, *S. jasminoides*, and *B. glabra* plants drastically shortened the lifespan of the sand flies. However, they lived longer when the same plant was presented together with blood, which might be neutralized by blood feeding or could also be due to the amount they feed on as toxicity was dose dependent (Benelli and Pavela, 2018). The prolonged survival in this study was not because the insects avoided the plants when given together with the blood; this is because 85% of the flies provided with intact *P. hysterophorus* and blood fed on the plant based on our molecular analysis. The survival probability of flies fed with *P. hysterophorus* alone was higher than on water used as control but not as much as when supplied with either glucose or blood. Flies fed with blood, blood + sugar as well as blood in combination with intact *P. hysterophorus* plant had an increased survival rate compared to the other non-blood treatments, showing the role of blood in enhancing stable fly’s survival. These findings were in line with a similar observation done by using different flower cuttings and pollen from wild plants, which prolonged *Stomoxys* survival more than water control (Jones et al., 1985, 1992). We found that the maximum days stable flies survived was 35 days when fed on blood and 16 days when they feed only on sugar under laboratory conditions.

The failure of stable flies to lay eggs when fed only on sugar demonstrated that stable flies are members of anautogenous insects where blood is necessary for egg development (Müller et al., 2012). Autogeny also occurs in other hematophagous Diptera, such as tabanids, chrysops, and deer flies (Rockel, 1969; Anderson, 1971; Bosler and Hansens, 1974).

Although sugar feeding has long been suggested in stable flies (Taylor and Berkebile, 2008), to the best of our knowledge, this is the first evidence directly linking plant feeding to stable flies’ fitness cost. According to our study, 3.67% of wild nectar feeding in stable flies was observed. Nectar feeding prevalence seems to be affected by the season and places of collection, for instance, Jones et al. (1992) reported higher nectar feeding from beach sites, which is 23%, but only 3% from dairy sites. Similarly, Taylor and Berkebile (2008) reported 8–12% nectar feeding that depends on season. Thus, the places and seasons of stable flies’ collection and detection methods might explain the variation. The availability and abundance of sugar sources determine the frequency of sugar feeding, with increased nectar feeding upon increased plant population (Martinez-Ibarra et al., 1997). Unlike male mosquitoes that die if deprived of sugar (Chadee et al., 2014), stable flies, both male and female, can survive without sugar for a long time but not without blood, showing that these two flies vary in their diet requirements for survival. Nectar feeding constitutes an important source of nutrition for adult mosquitoes of both sexes, particularly males, which feed exclusively from plant nectar and require frequent sugar intake for survival. This behavior is often underappreciated when compared with blood feeding, especially for stable flies as compared to mosquitoes or sand flies. The blood feeding behavior of stable flies is better studied (Mihok and Clausen, 1996; Getahun et al., 2020a) with more emphasis, most probably because it is during this process that transmission of pathogens can occur.

Our volatile organic compound analysis demonstrated that each plant has diverse odors. Furthermore, the plants were distinctly clustered into their respective family based on their chemistry. All the acacia trees clustered together, and the other plants were separated. Similarly, VOCs have previously been reported to potentially distinguish the closely related plant species, as observed in *Pinus mugo* species (Celiński et al., 2015). Each cluster was determined by a specific odor. However, the behavioral response of stable flies to those odors under field conditions varies from attractive (γ-terpinene and ocimene) to neutral or even repellent, i.e., caught significantly less as compared to that of the unbaited trap (butanoic acid, linalool oxide, methyl benzoate, etc.). The variation in behavioral response to those odors as predicted by SIMPER analysis shows that those odors might have a different role/or function. Furthermore, the absence of a strong attraction from some of the identified signature VOCs using SIMPER from the identified plants might suggest that additional volatile compounds or blends are necessary to induce attraction, as indicated by the significant attraction by the whole plant extracts. Furthermore, in addition to the smell of a plant, other features of a plant such as color, texture, shape, humidity, and local CO_2_ might play a significant role for plant acceptance (Burkle and Runyon, 2017; Nordström et al., 2017).

## Conclusion

The nectar feeding behavior of stable flies is governed by olfaction, and supplementing blood feeding with nectar feeding has fitness benefit and enhances egg fertility. The nectar source varies in the plant species that are diverse in their semiochemical composition. Thus, plant communities might affect the spatial and temporal distribution of stable flies’ populations and probably disease transmission dynamics either positively or negatively. Plant-derived VOCs can be exploited for the development of potential odor–bait technology, for instance, γ-terpinene to be used in vector surveillance and control. Plant nectar supplementation might also be recommended to boost stable fly laboratory colony.

## Data Availability Statement

The plants DNA sequence deposited in NCBI gene bank and the other data are presented in the article/Supplementary Material.

## Author Contributions

ST conceptualized and designed the experiment, collected and analyzed data, and wrote the manuscript. JB and DM supervised and proofread the manuscript. MG conceptualized the research idea and resource mobilization, designed and supervised the study, and prepared the manuscript. All authors contributed to the article and approved the submitted version.

## Conflict of Interest

The authors declare that the research was conducted in the absence of any commercial or financial relationships that could be construed as a potential conflict of interest.

## Footnotes

1 www.ncbi.nlm.nih.gov/BLAST/

2 www.sigmascientific.com
