# Supplementary material for: Supplementing Blood Diet With Plant Nectar Enhances Egg Fertility in Stomoxys calcitrans
Source: Front Physiol. 2021 Mar 30;12:646367. doi: 10.3389/fphys.2021.646367 (PMC8042263; doi:10.3389/fphys.2021.646367)
Supplement: Supplementary file 1 [file Table_1.DOCX]

| **VOC ID** | **VOC name** | **VOC ID** | **VOC name** | **VOC ID** | **VOC name** |
| --- | --- | --- | --- | --- | --- |
| Voc1 | Toluene | Voc31 | Caryophyllene<beta> | Voc60 | 3-Heptanone,_2-methyl- |
| Voc2 | Butylated_hydroxytoluene | Voc32 | Pentanoic_acid | Voc61 | Trimethyl_benzene<1,2,4-> |
| Voc3 | Tricyclene | Voc33 | Methyl_salicylate | Voc62 | Benzeneacetaldehyde |
| Voc4 | Ocimene<(E)-beta-> | Voc34 | Dendrolasin | Voc63 | Acetophenone |
| Voc5 | Ocimene<(Z)-beta-> | Voc35 | 3-Penten-2-one | Voc64 | Butanoic_acid |
| Voc6 | E-Ocimenol | Voc36 | decane | Voc65 | Pinene<alpha-> |
| Voc7 | Menthatriene<1,3,8-para-> | Voc37 | Dodecane | Voc66 | Pinene<beta-> |
| Voc8 | Linalool_oxide<cis->(pyanoid) | Voc38 | Sabinene | Voc67 | Hepten-2-one<6-methyl-5-> |
| Voc9 | Linalool_oxide_<trans->_(furanoid) | Voc39 | Selinene<alpha-> | Voc68 | Eicosane |
| Voc10 | Linalool | Voc40 | Selinene<beta-> | Voc69 | Tridecane |
| Voc11 | Cresol<para-> | Voc41 | Sabinene_hydrate | Voc70 | Methyl citronellate |
| Voc12 | Farnesene<alpha-> | Voc42 | Citronellic_acid | Voc71 | Hexenyl valerate<3Z-> |
| Voc13 | Farnesene<beta-> | Voc43 | Carene | Voc72 | Hexenyl butanoate<3Z-> |
| Voc14 | 3-Hexanone | Voc44 | Indole | Voc73 | Copaene<alpha-> |
| Voc15 | Ethyl_isovalerate | Voc45 | Nerolidol<Z-> | Voc74 | Copaene<beta-> |
| Voc16 | Hexanoic_acid<2-methyl-> | Voc46 | Nonane | Voc75 | Cedrene<alpha-> |
| Voc17 | Phellandrene<alpha-> | Voc47 | Hexadecane | Voc76 | Cedrene<beta-> |
| Voc18 | Phellandrene<beta-> | Voc48 | Tetradecane | Voc77 | Cinnamyl alcohol<E-> |
| Voc19 | Sesquiphellandrene | Voc49 | Heptadecane | Voc78 | Gurjunene<alpha-> |
| Voc20 | Anisole<para-methyl-> | Voc50 | Pentadecane | Voc79 | Gurjunene<gamma-> |
| Voc21 | Terpinolene | Voc51 | Undecane | Voc80 | Nerolidol<E-> |
| Voc22 | Camphene | Voc52 | Tritriacontane | Voc81 | Methyl jasmonate |
| Voc23 | 6-Camphenol_ | Voc53 | Docosane | Voc82 | Methyl_epi_jasmonate |
| Voc24 | Camphor | Voc54 | Eudesmol<10-epi-gamma-> | Voc83 | Farnesol |
| Voc25 | Myrcene | Voc55 | Amorphene<alpha-> | Voc84 | Farnesal |
| Voc26 | Tricosane | Voc56 | Amorphene<delta-> | Voc85 | Limonene |
| Voc27 | Methyl_benzoate | Voc57 | Cubebene | Voc86 | Cadina-1(6),4-diene<cis-> |
| Voc28 | Ethyl_benzoate | Voc58 | Dibutyl_phthalate | Voc87 | Germacrene_D |
| Voc29 | Phenyl_ethyl_alcohol | Voc59 | Trimethyl-1,3(E),7(E),11-tridecatetraene | Voc88 | Germacrene_B |

**Supplementary table : Volatile organic compounds identified from various plants stable flies fed on**

| **VOC ID** | **VOC name** | **VOC ID** | **VOC name** |
| --- | --- | --- | --- |
| Voc89 | Bicyclogermacrene | Voc129 | Dauca-5,8-diene |
| Voc90 | allo-Aromadendrene | Voc130 | alpha-Thujene |
| Voc91 | Isovalencenol<(E)-> | Voc131 | Cumacrene |
| Voc92 | Decene | Voc132 | cis-3-Hexenyl_isovalerate |
| Voc93 | Hexadecene | Voc133 | Benzaldehyde |
| Voc94 | Apofarnesal<(E)-dihydro-> | Voc134 | Sulfurous acid |
| Voc95 | Silphinene | Voc135 | Hexenyl isobutanoate |
| Voc96 | Octadecane | Voc136 | Tetracosane |
| Voc97 | Nonadecane | Voc137 | Cadinene<delta-> |
| Voc98 | Heneicosane | Voc138 | Hexadecane |
| Voc99 | 3-Phenylpropanol | Voc139 | Curcumene<gamma-> |
| Voc100 | Octane | Voc140 | Curcumene<beta-> |
| Voc101 | O-xylene |  |  |
| Voc102 | Mesitylene |  |  |
| Voc103 | Hexenyl acetate<3Z-> |  |  |
| Voc104 | Styrene |  |  |
| Voc105 | Sylvestrene |  |  |
| Voc106 | Naphthalene |  |  |
| Voc107 | Benzyl alcohol |  |  |
| Voc108 | Benzene acetaldehyde |  |  |
| Voc109 | Humulene |  |  |
| Voc110 | Humulene<alpha-> |  |  |
| Voc111 | Zingiberene<alpha> |  |  |
| Voc112 | Y_langene |  |  |
| Voc113 | Cymene<ortho> |  |  |
| Voc114 | Elemene |  |  |
| Voc115 | Elemene<gamma-> |  |  |
| Voc116 | Elemene<delta-> |  |  |
| Voc117 | Cymene<para> |  |  |

Supplementary table cont--
